# The Mental Health of Children with Cerebral Palsy: A Review of the Last Five Years of Research

**DOI:** 10.3390/jcm14124364

**Published:** 2025-06-19

**Authors:** Rebecca Rausch, Summer Chahin, Caroline Miller, Lindsey Dopheide, Nicholas Bovio, Ann Harris, Dilip Patel

**Affiliations:** 1 Homer Stryker M.D. School of Medicine, Western Michigan University, Kalamazoo, MI 49008, USA; 2 Doctoral Program in Clinical Psychology, Fielding School of Psychology, Graduate University, Santa Barbara, CA 93105, USA; 3 College of Education and Human Development, Counselor Education and Counseling Psychology, Western Michigan University, Kalamazoo, MI 49008, USA

**Keywords:** cerebral palsy, children, psychiatric disorders, mental disorders, mental health, academic performance

## Abstract

Background/Objectives: Children and adolescents with cerebral palsy (CP) often experience associated functional limitations, diseases, or impairments. Included in these associated concerns are mental health symptoms/disorders and academic concerns. There has been an increasing research focus on the mental health of youth with CP over the past 5 years, and there is a need to synthesize this research. This review aims to synthesize the most recent research on the mental and behavioral health of youth with CP. Methods: A literature search on research focused on mental health, academic functioning, and mental and behavioral treatment for youth with CP was conducted in August of 2024 and limited to the last 5 years to highlight the most recent developments in this area of research. Four hundred and forty-eight articles were screened, and thirty-eight articles were included in this review. Results: Based on this literature review, children with CP have high rates of mental health diagnoses across multiple diagnostic areas, including autism spectrum disorder, attention-deficit hyperactivity disorder, intellectual developmental disorder, anxiety, and depression. Academic concerns are common for children with CP. Intervention studies have focused on both child and parent interventions. Conclusions: Research over the past 5 years has added to prevalence estimates of mental health disorders in the pediatric CP population. Considering the high rates of mental health symptoms found in children with CP, future research should focus further on mental health interventions for this population.

## 1. Introduction

Cerebral palsy (CP) is a lifelong neurological disorder that originates in childhood, characterized by motor impairments affecting muscle tone, movement, and posture [1]. It is the most common physical disability in childhood, affecting approximately 1 in 500 live births worldwide [1]. Typically, CP presents in infancy and is strongly associated with preterm birth and perinatal complications. The condition is highly variable in its presentation and outcomes, influenced by the timing, location, and extent of brain injury sustained in utero or during early development. In addition to motor impairments, CP is often accompanied by a range of coexisting conditions, including cognitive, sensory, communicative, and behavioral challenges, as well as epilepsy and secondary musculoskeletal complications [1,2]. These factors significantly impact activities of daily living, frequently necessitating comprehensive, multidisciplinary care.

Recent studies have brought attention to the elevated risk of mental health symptoms or disorders among children with CP, emphasizing the need for further research in this area. A 2018 systematic review and meta-analysis highlighted the widespread but under-recognized prevalence of mental health symptoms in this population, with a pooled prevalence of mental health symptoms being found to be 35% and 28% in children with CP based on results of two parent-reported mental health screening tools, urging a deeper investigation into these issues [3]. Psychiatric conditions are common, with estimates suggesting that over half of children with CP meet diagnostic criteria for a psychiatric disorder by school starting age [4] and a significant proportion of these children experience psychological symptoms or social impairment [5]. Indeed, both attention-deficit/hyperactivity disorder (ADHD) and autism spectrum disorder (ASD) appear to be more common in youth with CP when compared to rates found in the general population [6]. The presence of depression and anxiety symptoms at a clinical level has been found in just over 30% of a sample of adolescents and young adults with CP [7]. This population may experience psychological and behavioral symptoms from a young age, with substantial behavioral and emotional symptoms documented in a large proportion of preschool children with CP [8].

The prevalence of sleep disturbances, intellectual impairment, and academic challenges further complicates the lives of children with CP. Similarly, intellectual disability (ID) is frequently seen in those with CP [9], and the presence of ID in this population is associated with an increased risk of psychiatric symptoms [3]. Academic underachievement, related to a combination of intellectual, speech, and neuropsychological problems or deficits, is a persistent challenge for many children with CP, further affecting their quality of life and long-term prospects [9].

Despite these widespread mental health and behavioral challenges, there is a paucity of effective, evidence-based interventions tailored to the specific needs of children with CP. Parenting interventions targeting behavioral problems have shown promise, with a randomized controlled trial demonstrating significant improvements in managing these issues [10]. However, many unmet needs remain, particularly in addressing the broader spectrum of mental health conditions and academic struggles. Holistic approaches that incorporate early identification, targeted therapies, and comprehensive family-centered care are critical for improving outcomes.

This review synthesizes the latest research published in the past five years on the mental and behavioral health of children with CP, with an additional focus on academic functioning and intervention strategies. The last review on this subject that we are aware of focused only on the prevalence of mental health concerns, included only eight studies, and did not focus on ADHD or ASD as mental health disorders [3]. A significant number of research studies on this subject have been published since this time. This paper aims to provide a more thorough narrative review of mental health concerns seen in youth with CP (including ADHD and ASD) and expand beyond prevalence to additionally include research focused on academic functioning, in addition to intervention studies. By examining recent findings on the prevalence, nature, and treatment of mental health challenges, this article aims to provide a comprehensive understanding of the difficulties faced by children with CP and to inform future efforts to improve their quality of life and overall well-being.

## 2. Materials and Methods

**Figure 1:**
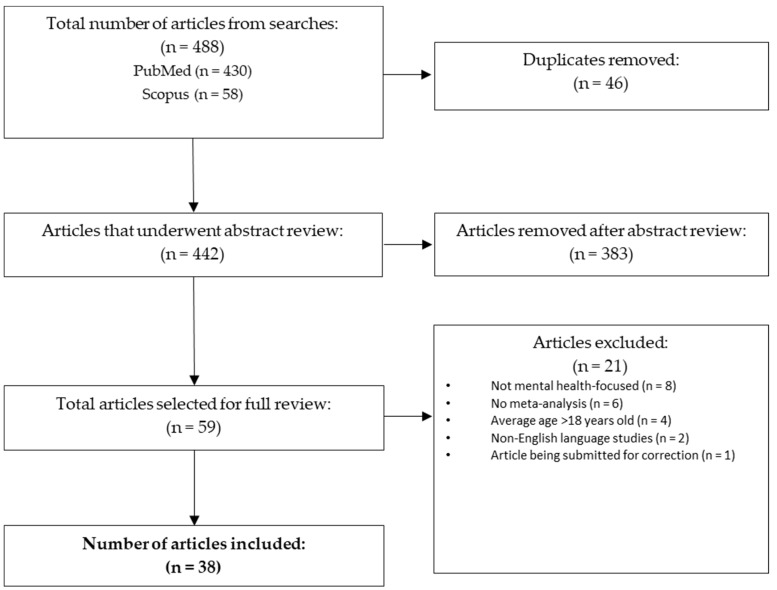
Flow diagram of article selection.

We conducted database searches in PubMed and Scopus for relevant literature from 2019 to August 2024 in order to focus on the most recent research literature on children with CP. Inclusion criteria were as follows: 1. research studies focused on children with CP; and 2. studies focused on mental or behavioral health (mental health disorders or symptoms), interventions for mental health disorders or symptoms, or academic functioning/learning disorders. We excluded studies based on the following criteria: 1. average age of participants was 18 or older, or data from participants was not collected from the child, adolescent, or youth age range (0–18 years old); 2. non-English language studies; 3. systematic reviews that did not include a meta-analysis; and 4. case studies. All articles were screened by the first author of this manuscript, and the first author utilized the inclusion and exclusion criteria to select finalized articles to be included in this review. The specific search strings are available in Appendix A. After removing duplicate articles, article abstracts were first reviewed and selected based on the inclusion criteria. A full review was conducted on selected articles, and based on further screening with exclusion criteria, 38 studies were chosen to be included in this literature review. Appendix B contains a table that reviews all articles included. Figure 1 presents the flow chart of article selection. Articles were organized by overall prevalence and trajectory of mental health disorders, diagnostic categories, academic functioning/specific learning disorders, and psychological interventions.

## 3. Results

### 3.1. Overall Prevalence and Trajectory of Mental Health Disorders

Three studies provided information on the overall prevalence of mental health diagnoses for children with CP when compared to typically developing peers [11,12,13].

A comprehensive study analyzing National Survey of Children’s Health data revealed that young individuals with CP face a significantly higher risk of developing various psychological disorders compared to their peers without CP [11]. The authors reported that children with CP had a higher prevalence of all mental health disorders examined and multimorbidity compared to children who did not have CP. After accounting for sociodemographic variables, the odds of mental health disorders in children with CP remained significant (except for ADHD), with reported odds ratios between 2.7 and 7.1 for included disorders [11]. After controlling for physical risk factors (including the presence of pain, sleep duration, and physical activity), the odds of anxiety and behavioral concerns remained elevated for children with CP, while the odds of depression were no longer significant for these children. However, these physical aspects only partially explained the higher prevalence of mental health disorders, indicating that other factors specific to CP may also contribute [11].

Rackauskaite and colleagues’ study [13] revealed a significantly higher prevalence of mental disorders in children with CP (22.4%) compared to controls (6.3%). ID was the most common mental disorder in the CP group, but even after excluding the presence of ID, children with CP still had a higher prevalence of other mental disorders [13]. Another study using the National Survey of Children’s Health data reported that children with CP in their sample had a higher overall prevalence of mental health disorders compared to typically developing peers, with reported rates of 75.5% of children with CP having a mental health disorder, compared to 54.2% of typically developing peers [12].

Researchers also reported information on the trajectory of mental health disorders for children with CP. Bjorgaas et al. [14] assessed a cohort of children with CP using a child psychiatric diagnostic instrument at both ages 7 and 11, with parents as informants. They found a significant increase in the prevalence of emotional disorders (which included anxiety and affective disorders) from 7 to 11 years of age, while the prevalence of behavioral disorders (encompassing conduct disorder, oppositional defiant disorder, and hyperactivity/attention deficit disorders) remained stable [14]. Half of the cohort met the criteria for a psychiatric disorder at both assessment points. The study revealed that subthreshold psychiatric disorders at age 7 were predictive of psychiatric disorders at age 11 [14]. In a study that sought to validate a screening measure and examine differences across informants, Bjorgaas et al. [15] also reported that the rates of mental health symptoms increased across childhood in a sample of children with CP.

### 3.2. Autism Spectrum Disorder

Ten studies included information on ASD [13,16,17,18,19,20,21,22,23,24] The prevalence of ASD within the pediatric CP population ranged from 3.4–30% [13,16,17,18,19,20,22,24]. A variety of reporting methods were used to assess for ASD across the studies. Parent-reported ASD prevalence rates resulted in rates of 6.09% [24], 11% [22], and 12.5% [20]. Higher prevalence rates were mostly reported in studies that utilized medical or registrar records of formal diagnoses, with rates of 3.4% [13], 18% [18], 20% [19], and 22% (reported to be taken from both medical records and parent reports) [23]. Lastly, ASD prevalence based on neuropsychiatric assessment was reported in two studies, with a rate of 30% [17], and 30% again in a subset of this same population [16].

Odds ratios calculated in multiple studies over the past 5 years highlight the association between CP and ASD. While sociodemographic variable-adjusted odds ratios for ASD in the pediatric CP population compared to typically developing peers were reported in two studies to be at 2.5 [13] and 2.97 [22], the highest adjusted odds ratio was reported at 5.07 [24]. In a study screening a population-based sample of children with CP, nearly 35% of participants screened positive for ASD symptoms [19]. This screening rate was almost twice as high as established clinical diagnoses of ASD, which were registered in only 20% of the sample [19], and another study found ASD symptoms in 48% of participants using another measure [20]. The presence of additional impairments (ID, higher motor impairment, greater speech impairment, and epilepsy) is associated with increased screening positivity for ASD, but increased additional impairments were also associated with a decrease in the number of established clinical diagnoses of ASD [19]. Diagnostic difficulties may occur for children with increasing impairments, leading to a need for more appropriate screening tools for children who meet this clinical picture [19]. One study found that while the average age of diagnosis for children with ASD alone was 4.92 years, children with CP were diagnosed with ASD at an average age of 5.95 years, demonstrating this gap [21].

The association between motor functioning and ASD status in children with CP was explored by studies. Rackauskaite et al. [13] did not find a significant association between motor function and ASD diagnosis. One study found decreasing frequency of ASD from Gross Motor Function Classification System (GMFCS) levels II to IV [19], and GMFCS level I and II encompassed the majority of the children diagnosed with ASD in Pahlman and colleagues’ [18] study. Utilizing a subset of participants from their 2020 project [19], Påhlman et al. [17] conducted further neuropsychiatric assessment and reported that a high proportion of new ASD diagnoses were found within participants at GMFCS levels III-IV. ASD was less prevalent in GMFCS level I than in levels II-V for their entire sample, but gross motor functioning was not associated with ASD itself [17].

Regarding CP type, Påhlman et al. [17] reported that while ASD was seen within all CP types, participants with ataxic CP frequently presented with comorbid ASD and ADHD, and the authors found a non-significant trend for spastic-type CP, with ASD being more common in those with bilateral spastic CP. ASD may also be more prevalent in children with CP who have predominant white matter injury when compared to other neuroimaging patterns [16]. While there was no difference in ASD frequency between unilateral and bilateral lesions, for participants with white matter injury, ASD was reported to be more common in those with bilateral lesions, compared to unilateral lesions [16].

These last five years of research also highlighted associated characteristics or commonly seen diagnoses in children with CP and comorbid ASD. Preterm or extremely preterm birth has been associated with this diagnostic profile [17,18,19]. The diagnosis of ASD within children with CP is predicted by both ID and ADHD [17]. More severe ID (when excluding profound ID) has been associated with increased frequency of ASD diagnosis for this population [18,19]. Sleep problems occur in children with ASD and CP, and those with comorbid ASD and CP; however, those with comorbid CP and ASD may experience a greater number of sleep problems than children with CP alone [21]. Consistent with an ASD diagnosis, children with comorbid ASD and CP also experience more social communication difficulties compared to children with CP and no comorbid ASD [21]. ASD symptoms in children with CP may also be associated behavioral problems and internalizing/externalizing symptoms [20].

### 3.3. Attention-Deficit/Hyperactivity Disorder

Thirteen studies included information on ADHD [11,12,13,16,17,18,19,20,22,24,25,26]. Prevalence rates of an ADHD diagnosis were reported in twelve studies [11,12,13,16,17,18,19,20,22,24,25,26], with a range of 4.1–31.0%. Prevalence of ADHD was reported by the different studies using a variety of identification methods. Utilizing National Health Interview Survey results, Chen et al. [24] reported that 15.91% of children with CP in their sample were diagnosed with ADHD, compared to 7.89% of children without CP, indicating that children with CP were at doubled risk for the diagnosis. Similar rates of ADHD were seen within samples over the last five years, with Casseus and Cheng [22] reporting 15% of children in their sample having ADHD compared to 8.7% of children without CP; Whitney et al. [25] and Whitney et al. [11] reporting a prevalence of 19.5%; Leader et al. [20] reporting that 15.4% of their sample was found to have a diagnosis of ADHD; and Cribb et al. [12] reporting that 19.9% of children with CP were diagnosed with ADHD, compared to 10.5% of typically developing peers. All of these studies [11,12,20,22,24,25] were based on parent reports of whether their child had the diagnosis, without direct assessment. Several studies reported ADHD prevalence rates based on electronic health records or medical/registrar records, with reported rates of 4.1% [13], 18.1% [26], 21% [18], and 23% [19]. Påhlman et al. [17] utilized comprehensive screening and neuropsychiatric assessments to assess a sample of children with CP and reported an ADHD prevalence of 30% and, in a subset of the same sample, reported a prevalence of 31% [16].

Odds ratios were also reported in multiple studies comparing rates of ADHD in children with CP to typically developing peers. After adjusting for age, sex, race/ethnicity, highest family education level, family income level, and geographical region, the odds ratio for children with CP (when compared to those without CP), was 1.95 for ADHD in one study [24]. Similar odds ratios were reported for children with CP compared to typically developing children by Cribb et al. [12] after controlling for sociodemographic variables, with an odds ratio of 2.1. Rackauskaite et al. [13] reported an ADHD odds ratio of 2.0 for children with CP, compared to children without CP, after adjusting for social variables. However, when compared to controls, Whitney et al. [11] reported that that the odds of ADHD were no longer increased after controlling for sociodemographic variables, and Whitney et al. [25] reported that the odds of ADHD were no longer increased after controlling for sociodemographic variables and the presence of pain.

Screening for ADHD symptoms was focused on by Påhlman et al. [19], with 50% of their sample screening positive for ADHD. This is an interesting result, as 23% of this sample had an existing, registered diagnosis of ADHD, indicating that more than twice as many children screened positive for ADHD when compared to the number of established diagnoses [19].

Studies conducted over the past five years have explored if CP type is associated with rates of ADHD diagnosis. Children and young adults with a diagnosis of ADHD in Casseus et al.’s study [26] were less likely to have bilateral spastic CP compared to peers without ADHD, with an adjusted odds ratio of 0.58 for those with bilateral spastic CP. Consistent with this finding, another study [18] found that unilateral spastic CP was more frequent in children with an ADHD diagnosis, compared to those with bilateral spastic CP. CP type varied within participants diagnosed with ADHD, with 27% having unilateral spastic CP, 12% having bilateral spastic CP, 16% having dyskinetic CP, and 44% having ataxic CP [18]. Those with ataxic CP often presented with a combination of ADHD and ASD in Påhlman and colleagues’ study [17], with a significant overrepresentation of ADHD. Lastly, ADHD was seen more often in children who experienced a middle cerebral artery infarction, with 62% displaying this pattern compared to 28% with a different pattern [16]. The prevalence of ADHD was not different between participants with unilateral and bilateral lesions [16].

GMFCS level was frequently associated with rates of ADHD within these studies. Children with GMFCS levels III-V, indicating more functional limitations, had a lower prevalence of ADHD compared to children with GMFCS levels of I-II, at 15.9% and 62.2%, respectively [26]. Children with a GMFCS level between III-V had lower odds of an ADHD diagnosis, with an adjusted odds ratio of 0.10 [26]. Påhlman et al. [19] found a decrease in the number of identified diagnoses of ADHD from GMFCS level II to V, and this was mirrored by another study [17] reporting that ADHD was less prevalent in GMFCS levels IV-V. Further highlighting lower rates of ADHD at higher GMFCS levels, GMFCS levels I and II were most common for children diagnosed with ADHD in Påhlman et al.’s study [18]. However, Rackauskaite et al. [13] reported that there was no significant association between ADHD and the level of motor functioning.

Studies also examined a number of comorbidities and characteristics associated with ADHD in children with CP. Twenty-nine percent of children had an overlap in screening positivity of ADHD and ASD symptoms in a screening study [19], and ADHD was comorbid with ASD in 7% of Påhlman and colleagues’ sample [18], in addition to this research group’s 2022 study reporting a comorbidity rate of 15% [16]. One study found that the presence of ADHD was predicted by ASD [17], with an odds ratio of 3.0. ID was associated with ADHD in Casseus et al.’s [26] sample, and the diagnosis of ADHD was predicted by ID (odds ratio of 2.3) in another sample [17]. Those with more severe ID (when excluding those with profound ID) more often screened positive for ADHD, and identified ADHD diagnoses were shown to be more prevalent in children with less severe ID [19]. ADHD was rarely diagnosed in children with severe ID, and no children with profound ID were reported to have comorbid ADHD [18].

The trajectory of diagnoses was examined by one study, which found that the trajectory of behavioral disorders, of which ADHD/ADD was the most common, was stable across the study interval (spanning the ages of 7 years old and 11 years old) [14].

### 3.4. Intellectual Developmental Disorder (Intellectual Disability)

Ten studies included information on ID [13,16,17,18,19,20,21,23,27,28]. The prevalence rates of a diagnosis of ID were reported in nine of the studies [13,16,17,18,19,20,21,23,27] and ranged from 10.8 to 59%. ID frequently co-occurs with ASD and ADHD in children with CP. Pahlman et al. [17] found that 51% of their CP sample had an ID, with ID emerging as the strongest predictor of ASD and ADHD. In a subsequent study, Pahlman et al. [19] found that 59% of children with CP had ID, with severe ID (IQ from 20 to 34) linked to higher ASD prevalence and ADHD being more common in those with milder ID. Additionally, Pahlman et al. [16] found that ID was present in 54% of their population and determined that ASD was more common when there was a comorbid diagnosis of ID. Pahlman et al. [18] reported a prevalence rate of 53% for their CP population and further emphasized that ASD prevalence increased with ID severity, while ADHD was rarely diagnosed in severe ID cases. Cummins et al. [27] corroborated these findings, noting that ASD was present across all intellectual levels in spastic CP populations, and that its prevalence rose with increasing ID severity. The prevalence rate of ID reported in Cummins and colleagues [27] was 40.4% in their sample of individuals with spastic-type CP, although the authors indicated that a limitation of their study was ID being determined by clinician “impression,” rather than a standardized assessment procedure. Leader et al. [21] demonstrated that ID was a significant predictor of adaptive behavior impairments, indicating that co-diagnosis with ID exacerbates overall impairment in CP patients. Furthermore, Leader and colleagues [20] found that 58.7% of their population had ID and that the presence of ID (along with sleep problems, internalizing and externalizing symptoms, and ASD symptoms) predicted the presence of behavioral problems. De Clercq et al. [23] reported an ID prevalence of 26.3% in their sample, based on medical records. Rackauskaite et al. [13] identified that 10.8% of Danish children with CP in their sample had comorbid ID; however, the authors acknowledged potential underestimation due to diagnostic coding practices.

Perinatal factors were explored by Cummins et al. [27], who examined a sample of individuals with spastic CP and discovered that severe ID was more prevalent in infants born at term compared to preterm births. Furthermore, infants with birth weights below two standard deviations of the mean had a higher risk (36%) of severe ID compared to those with normative birth weights (26%) [27].

Pahlman et al. [16] reported that ID was more common in children with bilateral CP compared to other CP types. Additionally, ID was less prevalent in children with predominant white matter injuries but more frequent in those with middle cerebral artery infarctions [16]. Cummins et al. [27] found that children with bilateral spastic CP had a sevenfold increase in the odds of having a severe ID compared to those with unilateral spastic CP. Olusanya et al. [28] highlighted that global estimates of CP and ID often stem from high-income countries, suggesting that variations in CP type and associated brain injuries across different regions may influence ID prevalence rates.

The relationship between GMFCS level and ID prevalence in children with CP was explored. Pahlman et al. [18] observed a direct correlation between higher GMFCS levels and increased ID prevalence, noting that severe motor impairments were associated with higher rates of ID. Cummins et al. [27] reinforced this relationship, indicating that severe ID was strongly linked to higher GMFCS levels. Moreover, Pahlman et al. [19] noted the challenges in assessing children with GMFCS level V and profound ID (IQ < 20) due to inappropriate assessment tools.

Methodological constraints and regional disparities impacting the assessment and reporting of ID prevalence were noted in studies. Rackauskaite et al. [13] acknowledged that ID prevalence might be underreported due to data cleaning procedures and the secondary nature of ID diagnoses in clinical encounters. Cummins et al. [27] also pointed out potential underestimation issues related to assessment practices for severe ID. On a global scale, Olusanya et al. [28] emphasized that CP and ID prevalence rates are typically derived from high-income countries, potentially overlooking higher prevalence rates in low- and middle-income regions.

### 3.5. Anxiety, Depression, and Mood

Ten studies reported information on anxiety, depression, or both diagnoses for children with CP [11,12,13,14,22,25,29,30,31,32].

Separation anxiety symptoms in children with CP have been documented from a very early age [29], and anxiety is particularly pronounced in girls, who are 2.3 times more likely to be diagnosed with anxiety disorders compared to boys [30]. Researchers found that individuals with CP have a significantly higher risk of developing mental health diagnoses such as anxiety and depression compared to those with typical development [13]. Rackauskatie and colleagues [13] combined affective disorders, anxiety disorders, and OCD into one category, finding these conditions to be prevalent in a significant portion of the CP cohort, with reported rates of 4.8% in children with CP, compared to 2% of controls. An adjusted odds ratio (adjusted for social variables) was reported to be 2.7 in children with CP for these types of diagnoses [13]. After controlling for sociodemographic variables, Cribb et al. [12] reported adjusted odds ratios of 2.6 for anxiety and 1.8 for depression. In an observational study, between 15 and 30% of youth (youth- and caregiver-reported) experienced anxiety and depression symptoms that were elevated or very elevated [32]. Compared to controls, Whitney et al. [25] found that children with CP had higher prevalence rates of depression (7.8% of children with CP vs. 2.7% of controls) and anxiety (30.2% of children with CP vs. 6.2% of controls). Casseus and Cheng [22] found that a rate of 26.5% of their sample experienced anxiety, making it the second most commonly reported health condition reported by participants. With similarly high rates, another study found that significant anxiety was found in 46% of participants based on child reports and a lower number based on parent reports, at 38% [30].

However, one study reporting on a sample of adolescents and adults in urban South Africa found that despite children with CP having physical challenges, their mental health scores were not different from typically developing children and adolescents [31]. Researchers have suggested that there may be under-diagnosis of mental health concerns, especially anxiety, in the CP youth population [30,32]. McMahon and colleagues [30] found that anxiety was not recognized by medical providers in 43% of youth who were presenting with clinically significant anxiety after reviewing parent reports on the Screen for Child Anxiety Related Disorders clinical measure [30].

A variety of research has been conducted on the experiences of pain, quality of life, and mental health and the relationship with CP [11,31]. Findings indicated that individuals with CP often experience significant pain and lower health-related quality of life compared to the general population. Researchers aimed to identify factors influencing these outcomes in a socioeconomically diverse setting. The results of this study highlight the intersection of chronic pain, mobility limitations, and mental health challenges that individuals with CP face in a resource-limited environment [31]. Researchers also found that a higher prevalence of chronic pain among adolescents with CP correlated with lower health-related quality-of-life scores [31] However, even when accounting for physical risk factors, children with CP may have higher rates of mental health diagnoses [11]. Whitney et al. [11] found that the relationship between CP and depression was at least partially accounted for by physical activity and pain.

The relationship between physiological symptoms such as pain and fatigue, and mental health symptoms such as anxiety and depression, in youth with CP were examined [32]. Results from this study indicated that physiological symptoms such as fatigue, muscle pain, and sleep disturbances often co-occur with mental health symptoms in youth with CP, which suggests a bi-directional relationship between physical and mental well-being [32]. Further, Testani and colleagues’ research [32] indicated that 85% of youth with anxiety disorders experience at least 1 sleep issue, and that approximately 55% experience three or more sleep-related issues, further complicating their mental health. Fatigue and sleep problems (such as restless sleep or challenges with falling asleep) also play critical roles, with fatigue identified as a strong predictor of both depression and anxiety symptoms [32]. These sleep issues combined with lower quality of life are also further associated with higher risk of developing mental health concerns, both in the short and long term, and impact general engagement in day-to-day activity [32].

The relationship between mental health, physical activity, and sports participation among children with CP and how this relates to anxiety and depression was examined. Cribb and colleagues [12] explored how physical activity, including sports, might influence mental health well-being in children with CP, as well as barriers to participation. A positive correlation between physical activity and better mental health outcomes was found, as well as reduced symptoms of both anxiety and depression [12]. Participation in sports was also further found to be associated with increased self-esteem, improved mental health, improved physical function, improved social interactions, and improvements in overall well-being [12]. However, there remain several barriers to engagement in sports for youth with CP, including limited access to specialized programs, financial constraints, and physical barriers that hinder full participation in sports and physical activity [12].

Further, Whitney and colleagues [25] explored the role of participation in activities and experiences of bullying in relation to mental health in children with CP. Their research explored how these factors interact to affect the emotional well-being of children with physical disabilities. Highlighting the impact of modifiable factors on the mental health of children with CP, Whitney et al. [25] reported that children with CP had a higher prevalence of depression and anxiety compared to children without CP. When examining the adjusted odds ratio of these diagnoses in children with CP, after controlling for sociodemographic variables and the presence of pain, the odds of anxiety remained statistically significant, while the odds of depression were no longer significantly increased. Authors reported that the relationship between CP and anxiety was accounted for by difficulty with friendships [25].

Evidence suggests that increased participation in physical activities can mitigate the risk of depression in these children [32]. However, researchers have found that there is increased risk of developing depression symptoms in youth with CP when they also experience pain, and the experience of fatigue was a predictor related to youth-reported depression and anxiety scores [32].

Regarding the trajectory of these diagnoses, researchers conducted a longitudinal study and tracked the development of psychiatric disorders in children with CP over a 4-year period [14]. Authors found that youth with CP demonstrated a four-fold increase in emotional disorders between the ages of 7 and 11 [14]. Authors also reported an association between the presence of behavioral disorders at age 7 and the presence of emotional disorders at age 11, positing that early behavioral disorders may be a risk factor for later emotional symptoms [14].

### 3.6. Behavioral Concerns and Disorders

Nine studies examined behavioral concerns and disorders in youth with CP [11,12,14,20,22,23,25,33,34].

Behavioral challenges or disorders are prevalent in children with CP, with approximately 25.6% exhibiting significant behavioral issues [34]. Consistent with this number, Cribb et al. [12] reported that 24.6% of their sample of children with CP had a behavioral disorder, and Whitney et al. [25] and Whitney et al. [11] reported behavioral problems occurring in 27.3% of their sample. Another study found that based on parent reports, 20.2% of children with CP have behavioral or conduct problems, compared to typically developing peers, with a reported rate of 7.0% [22], while Leader et al. [20] reported a very high prevalence of behavioral concerns in their sample of children with CP, at 88.5%. The highest reported behavioral concern was stereotyped behavior, which occurred in 74% of their sample, followed by self-injurious behavior (73%) and aggressive/destructive behavior (57.7%) [20]. Compared to typically developing peers, children with CP are more likely to exhibit behavioral concerns/meet criteria for a behavioral disorder, with an odds ratio of 4.8 (controlling for sociodemographic variables) [12]. An adjusted odds ratio of 3.9 (controlling for sociodemographic variables and chronic pain) for behavioral problems in children with CP was reported by Whitney et al. [25].

The most common behavioral challenges include issues with peers and emotional dysregulation [34]. These problems are often compounded by sleep disturbances and nighttime pain, which are linked to increased behavioral difficulties [34]. The rates of behavioral concerns in children with CP are about double those found in typically developing peers, even as early as the pre-school years [34]. Horwood and colleagues [34] found that the frequency of conduct and emotional issues were even higher in the school-aged group of children with CP compared to the preschool-aged group of children with CP and reported that behavioral difficulties, including irritability and social withdrawal, were common [34]. Within this sample, sleep problems, particularly difficulty falling asleep and frequent awakenings, were reported often [34]. Additionally, nighttime pain was another significant issue impacting the well-being of children with CP, which was found to potentially exacerbate both behavioral and sleep disturbances [34].

Gardiner and colleagues [33] examined the different types and patterns of behavioral problems in children with neurodevelopmental conditions, such as CP, and identified overlaps between different disorders. They sought to categorize various behavioral problems, such as hyperactivity, anxiety, and aggression, and explored how these different behaviors manifest differently in children with CP compared to other neurodevelopmental conditions. They also found that behavioral problems overlap significantly across these conditions, which suggests similar underlying mechanisms for children with CP, children with ASD, and children with global developmental delay/ID [33].

Examining the trajectory of mental health disorders in children with CP between the ages of 7 years and 11 years, Bjorgaas et al. [14] reported that the prevalence of behavioral disorders (including ADHD and oppositional defiant disorder) was stable between these two age groups. Behavioral issues may become more observable at an earlier age in children with CP. Interestingly, the presence of behavioral disorders at age 7 in children with CP has been associated with the presence of emotional disorders at age 11, indicating an important relationship that points to the need for early intervention focused on behavioral concerns [14].

Research over the last five years has focused on associated characteristics of behavioral concerns in children with CP. Leader and colleagues [20] investigated the relationship between complex comorbidities and harmful behaviors, such as aggression and self-injury, in youth with CP. They found that children with multiple co-occurring conditions such as CP, epilepsy, ID, and more were at a much higher risk for developing harmful behaviors. Specifically, ID, sleep problems, internalizing and externalizing behaviors, and ASD symptoms predict more challenging behavioral issues in children with CP [20]. Sleep concerns predicted not only the frequency, but also severity of stereotyped behavior, self-injurious behavior, and aggressive/destructive behavior [20].

In a study focused on the interaction of parenting style, child personality characteristics, and psychosocial development over time, De Clercq et al. [23] found that parenting styles stayed stable over a two-year period, but child psychosocial development changed over this period of time. Within this sample, both child personality factors and parenting behavior were risk factors for behavioral problems (but also protective factors for psychosocial strengths), highlighting the interaction of parenting and personality. Specifically, greater externalizing problems in youth with CP were associated with externally controlling parenting [23].

Modifiable factors related to behavioral concerns were identified by authors. Participation in daily physical activity or sports has been associated with a decreased likelihood of behavioral conditions [12], Difficulties with friendships and bully victimization have also been shown to account for some of the relationship between CP and behavioral concerns, with authors pointing to modifiable factors that may be able to improve behavioral functioning within this population [25]. Lastly, pain may also account for at least a portion of the association between behavioral concerns and CP [11].

### 3.7. Academic Difficulties and Learning Disorders

Six studies included information on academic difficulties or learning disorders [35,36,37,38,39,40]. Of these six studies focused on academic concerns, the prevalence rates of a diagnosis of ID were reported in only one study [38]. Two of the studies were assessments of the efficacy of educational programs on children with CP [39,40]. One study compared school outcomes of adolescents with CP to a general population comparison group [37], and one compared the numerical cognition of children with CP with the numerical cognition of typically developing children [36]. One study examined the impact of brain lesions on functioning, including academic functioning [35].

Micheletti and colleagues [38] reported that over half of the children with CP had at least one learning disorder. de Freitas Feldberg et al. [36] documented lower academic performance in arithmetic and other subjects when comparing children with CP to typically developing peers. In the sample of children with CP examined in Micheletti et al.’s study [38], 38.1% had multiple learning disorders, and the prevalence of various types of learning disorders (i.e., reading, writing, and mathematics) was comparable for the CP children and the group of children with SLDs (not diagnosed with CP).

Micheletti et al. [38] indicated that children with CP who also had learning disorders scored significantly lower on measures of working memory and visual–spatial intelligence, compared to children with CP who did not have learning disorders. de Freitas Feldberg et al. [36] found that children with bilateral CP, in particular, demonstrated impairments in working memory, positing that this may partly explain their challenges with numerical cognition. Laporta-Hoyos et al. [35] found that lesions on the medial dorsal thalamus, parietal lobe, and temporal lobe (lateralized to the left hemisphere) accounted for poorer verbal performance in children with CP. Children with bilateral lesions demonstrated better performance in verbal cognitive functions compared to children with unilateral left-sided lesions [35]. The authors reviewed that this pattern highlights the role of specific cognitive deficits, especially in working memory, visual-spatial processing, and verbal cognition in shaping the outcomes for children with CP, indicating potential areas for targeted cognitive interventions [35].

Examining an educational program for children with CP, Pereira et al. [39] found that a nine-week narrative-based intervention program designed specifically for this population led to improvements in behavioral, emotional, and cognitive engagement, suggesting that storytelling and group dynamics may enhance learning engagement. However, Wotherspoon et al. [40] observed no significant effects of a web-based cognitive rehabilitation program (Strengthening Mental Abilities through Relational Training) on the academic outcomes of children with mild to moderate CP, which they attributed to difficulties with engagement and program completion.

Two studies compared the educational outcomes of children with CP to those of typically developing peers or general population samples. Jarl and Alriksson-Schmidt [37] demonstrated that, even when controlling for ID and motor difficulties, adolescents with CP scored lower on measures of school achievement than their peers. The researchers indicated that it is difficult to untangle the associations between communication difficulties, ID, and school outcomes for adolescents with CP [37]. Similarly, de Freitas Feldberg et al. [36] found that children with CP had significantly lower performance on cognitive tasks, especially in arithmetic and numerical cognition, compared to typically developing children.

### 3.8. Interventions for Children with CP

Ten studies examined recent therapeutic interventions designed to target various components of CP and associated sequelae in pediatric and adolescent populations [12,22,41,42,43,44,45,46,47,48]. Three studies examined caregiver targeted interventions and care [22,42,48]. Two studies examined an intervention delivered to both child and caregiver [44,45], and the remaining five studies examined therapeutic interventions delivered primarily to children to address behavioral problems [12,47], attentional problems [12,41,43], emotional problems [12], and physiological problems [46] secondary to CP.

#### 3.8.1. Parent-Focused Interventions

A randomized control trial by Whittingham et al. [48] evaluated the effectiveness of a ten-week Parenting Acceptance and Commitment Therapy (PACT) intervention, aimed at improving the emotional availability of the caregiver/child relationship, as well as improving caregiver psychological well-being and child psychological adjustment. Sixty-seven parents and caregivers of children with CP were recruited from various medical settings, the Australian Cerebral Palsy Register, and through word of mouth [48]. Caregivers were randomly assigned to either the PACT intervention or a waitlist control group. The self-directed PACT intervention was delivered online to the experimental group over a 10-week period, with pre-test, post-test, and 6-month follow-up data collection periods. Two components of emotional availability significantly improved following PACT intervention when compared to the control group: parental non-intrusiveness and child involvement [48]. Group comparisons also revealed significant improvement for the PACT group in the following domains: parental mindfulness, parent reported child quality of life (participation/physical health and social wellbeing/acceptance), parental comfort with child CP diagnosis, parental likelihood to seek help from others, parental likelihood to maintain social connections, and parental meaningful living [48]. With the exception of social subscales of child quality of life, all intervention effects were maintained at 6-month follow-up [48]. The PACT intervention was not shown to have an effect on parental mental health, child behavior, or child psychological well-being [48].

A quasi-experimental study by Alibakhshi et al. [42] evaluated the effectiveness of a communication skills intervention program for improving mother–child interaction and diminishing child behavioral problems. Forty-two mothers of children with CP were randomly sorted into an experimental group or a control group [42]. Participants in the experimental condition received the communication skills intervention in eight 90-min sessions, delivered over the course of 8 weeks, while an independent blind observer evaluated child behavioral problems during mother–child interactions both pre- and post-intervention [42]. Significant improvement in behavioral problems was observed in the following domains: aggression and hyperactivity, social incompatibility, antisocial behavior, anxiety, and depression, as well as in general behavioral problems overall [42].

Using data from the National Survey of Children’s Health, Casseus and Cheng [22] sought to determine how effectively needs for multidisciplinary care coordination are being met for children with CP. Their sample included responses from 88,150 parents and caregivers, with the majority of responses representing caregivers of children without CP (n = 87,861) and the remaining representing those with children diagnosed with CP (n = 289) [22]. Children with CP had higher odds of having unmet care coordination needs than children without CP (aOR = 2.63), and children with moderate to severe CP had greater odds of unmet care needs than children with mild CP (aOR = 3.16). The odds of children with CP having unmet care coordination needs also increased for children with public insurance (aOR = 1.37), and for children with low household income (aOR = 1.13) [22].

#### 3.8.2. Parent- and Child-Focused Interventions

A randomized controlled trial by Mak et al. [44] evaluated the effectiveness of a mindfulness yoga program (“MiYoga”) at improving child attention and psychological well-being, as well as parental mindfulness and psychological well-being. This study specifically aimed at determining if intervention effects observed following the initial intervention pre-post analysis were maintained at a six-month follow-up. Twenty-three parent/child dyads participated in this analysis [44]. The MiYoga intervention consisted of six 90-min sessions, completed in 20 min daily intervals over the course of 8 weeks [44]. The initial benefits to child attention were not maintained at 6 months, and child attention at follow-up was also not significantly different than pre-intervention [44]. Analyses further revealed that child behavior did significantly improve between post-test and the 6-month follow-up, though no other improvements to child psychological or mindfulness outcomes were maintained [44]. Child improvement in executive functioning and physical functioning observed at post-test were maintained at 6 months. For parents, analyses revealed a significant improvement from post-test to the 6-months follow-up in personal well-being, though no other changes to parent psychological flexibility or well-being were maintained at 6 months [44].

Another study by Ödek et al. [45] examined the effects of Equine Facilitated Activities on various components of children and their mothers’ psychosocial functioning, including anxiety, depression, empathy, and aggression. Twenty child/mother dyads were recruited and randomly divided into experimental and control groups. Children in the experimental condition received Equine Facilitated Activities therapy in one-hour sessions over the course of 8 weeks. Analyses revealed significant time and group differences in child anxiety as reported by children and parents, in addition to aggression and empathy, for those in the experimental condition [45]. Significant time and group differences were also observed for mothers with those whose children were in the experimental condition reporting significantly lower rates of depression and trait anxiety than those with children in the control condition [45].

#### 3.8.3. Child-Focused Interventions

Three studies examined the effectiveness of therapeutic interventions on improving attention difficulties for children with CP. In a two-arm parallel randomized trial, Chen et al. [43] examined the effects of neurofeedback in children with CP and comorbid attention deficits. Of the 24 children, 12 were randomly assigned to receive the neurofeedback intervention, which was delivered through a video game format in one-hour sessions twice per week for 10 weeks, for a total of 20 sessions [43]. Analyses revealed a significant between-group difference in beta/theta ratio at the conclusion of the intervention [43]. While this outcome would suggest an improvement in attentional control, significant group differences were observed in only one component of the Conners CPT-II measure, showing some decrease in omission rates for the intervention group [43]. Finally, significant group differences were also observed for visuoperceptual skills, with the intervention group demonstrating significant improvement in visual sequential memory and visual closure compared to the control [43].

Another study examining the effectiveness of therapeutic interventions on attention is an experimental design study by Ahn and colleagues [41]. Researchers here specifically examined the effects of Equine-Assisted Activities (EAA) on attention and psychological well-being for children with CP. Forty-seven participants were recruited from a university hospital in Seoul, South Korea, fifteen of whom had a comorbid diagnosis of ADHD [41]. Children randomly assigned to the intervention group received EAA delivered in 40-min sessions, twice per week, over 16 weeks (32 EAA sessions total) [41]. While many aspects of attention measured on the Conner’s CPT-II test improved with time for the EAA group, these changes were not significantly different from the control group, with the exception of preservation error. For children in the ADHD subgroup, children in the EAA group demonstrated significant improvement in d’, commission, and perseveration score, though between-group comparisons in this subsample were not reported [41]. Finally, while significant improvement in quality of life was observed over time for the EAA group, these changes were not significantly different at post-test from the control group [41].

A third study included changes in attention as one of several outcome measures. Using data from the National Survey of Children’s Health, Cribb et al. [12] examined the relationship between physical activity and sports participation with anxiety, depression, ADHD, and behavioral problems in children with CP. Data from children between the ages of 6–17 with CP (n = 458) was compared to data from typically developing children (n = 40,091) [12]. Results demonstrate a significant decrease in the likelihood that a child with CP will experience anxiety (OR = 2.2), depression (OR = 1.4), behavioral disorders (OR = 4.1), and ADHD (OR = 1.9), if they participate in sports [12]. Similar significant decreases in odds were observed in all domains for children with CP who engaged in daily physical activity. However, interaction effects demonstrated that sports participation and daily physical activity did not protect against mental health disorders for children with CP [12].

Additional studies included outcome measures specifically examining intervention effects on internal/emotional and external/behavioral experiences for children with CP. A quasi-experimental study by Shahriari et al. [47] examined the effectiveness of Child-Centered Play Therapy (CCPT) for children with CP. Recruited through convenience sampling, 30 participants were randomly and evenly divided into experimental and control groups. Children in the experimental condition received 16 45-min sessions, twice per week for 8 weeks [47]. Analyses revealed a significant reduction in anxiety, depression, offensive behavior, and aggression in the CCPT group, as was indicated on the behavioral symptoms list used as the sole outcome measure [47]. Significant group differences were also reported [47]. The authors report that data gathered at an unspecified follow-up stage demonstrated that the intervention effects in all domains were maintained at follow-up [47].

A final, population-based case-control study by Samjin et al. [46] examined the effects of incontinence training with urotherapy in addressing enuresis in children with CP. Both children with CP and typically developing children were recruited from the CP-Reference Center at the Ghent University Hospital, the Urology and Nephrology department at the Ghent University Hospital, and associated schools [46]. Forty-five children, ages 5–12, either with CP or typically developing, with daytime, or both daytime and nocturnal enuresis, received urotherapy for one year [46]. Although the procedure for delivering the urotherapy was standardized, after the first 3 months of treatment, specific interventions were individualized to the needs of each child. Analysis of data collected at pre- and post-intervention, as well as at 3-month intervals, revealed that incontinence training with urotherapy effectively reduced daytime enuresis, incontinence frequency, and fecal incontinence in children with CP, though these results were slower to occur, and the intervention was overall less effective when compared with the typically developing cohort [46].

## 4. Discussion

Research over the past five years focused on the mental health of children with CP highlights the significant risk of mental health concerns for this population. The studies reviewed in this paper add to the literature base regarding the prevalence of mental health diagnoses and symptoms, associated characteristics with mental health concerns, academic functioning, and interventions focused on mental or behavioral health for youth CP.

Previous research has indicated that children with CP demonstrate more emotional and behavioral symptoms when compared to siblings [49], and that a significant proportion of this population is at high risk for poor mental health [5]. While exact prevalence rates of mental health diagnoses in children with CP have not always been clear [3], studies published since August of 2019 indicate that this population has a higher prevalence of mental health disorders when compared to typically developing peers [12,13], even when accounting for physical risk factors [11]. The prevalence of mental health symptoms or diagnoses is clearly increased for children and adolescents with CP, and this heightened risk spans across multiple diagnostic categories.

Based on studies included in this review, the odds of ADHD [12,13,24], ASD [13,24], anxiety [12,25], depression [12], and behavioral concerns [12,25] were elevated when compared to typically developing peers. Previous research has indicated that over half of children with CP may meet diagnostic criteria for a mental health disorder by school-starting age [4]. Symptoms of mental health concerns at an early age are predictive of mental health concerns at a later age for this population [14]. Similarly to children, adults with CP are at a higher risk of mental health diagnoses [50,51]. Research has indicated that the psychological symptoms or social concerns of children with CP (such as attention, peer, and social interaction problems) may continue into adulthood [52]. This underscores the importance of early identification of mental and behavioral health concerns in children with CP and the importance of early intervention.

### 4.1. Specific Mental Health Concerns

While there was a large range of reported rates of ADHD in the studies reviewed, prevalence rates of this diagnosis in studies of children with CP over the last 5 years were primarily high. A 2023 systematic review on the global prevalence of ADHD reported rates of 7.6% in children (between ages 3 and 12 years) and 5.6% in teenagers (between ages 12 and 18 years) [53]. Only one of the studies reviewed in this paper on ADHD in children with CP [13] reported a prevalence rate lower than those for the global prevalence of ADHD in children and adolescents [53]. While two studies utilized psychological assessment methods [16,17], most studies utilized information from surveys, electronic medical records, or parent reports. These different modalities of data collection, in addition to different populations across the studies, likely led to this wide range of reported prevalence rates.

Children with CP are at increased risk for an ASD diagnosis compared to typically developing peers [13,22,24], and many display symptoms consistent with ASD [19,20]. A worldwide prevalence rate of ASD has been reported to be 0.6% [54]. The studies reviewed in the current paper reported higher rates of ASD prevalence in their samples of children with CP [13,16,17,18,19,20,22,24]. One study reported fewer ASD diagnoses in children from GMFCS levels II to IV [19]. Children with comorbid CP and ASD may also have a number of associated characteristics, such as preterm birth [17,18,19], ID [17,18,19], and sleep concerns [21]. Higher prevalence rates of ASD in children with CP (29–30%) were previously found in studies focused on medical conditions associated with CP, indicating that these conditions may increase the risk of ASD in the CP population [6]. Based on differences in screening rates and registered diagnoses of ASD within samples of children with CP [19] and delays in the timing of ASD diagnoses compared to children without CP [21], this population likely faces under-diagnosis of this disorder.

While there was a wide range of prevalence rates for comorbid ID and CP diagnoses, the research conclusions over the past five years suggest a high prevalence of ID in patients diagnosed with CP [13,16,17,18,19,20,21,27,28]. Previous reports on the prevalence of ID in CP populations have been reported to be 45% [55]. Additionally, GMFCS level was not often referenced when reporting the prevalence of ID within CP populations; however, Pahlman et al. [18] and Cummins et al. [27] suggested that ID is more strongly associated with severe GMFCS levels. This mirrors results in which GMFCS level and intelligence levels have been found to be significantly correlated, with increasing GMFCS levels being associated with lower intellectual abilities [56]. Pahlman et al. [19] noted that screening instruments used in their study were inappropriate for participants with GMFCS level V and profound ID, leading to a group with too few items being completed to be able to evaluate them. This highlights the need for finding additional tools that would allow for appropriate evaluation of these children.

Caution should be used regarding the results of IQ assessments for children with CP, as adaptations of intelligence scales for this population are not standardized [9]. Children with CP and more severe motor impairment often do not undergo adequate evaluation of their cognitive functioning [57]. These difficulties with assessment may contribute to a misrepresentation of the frequency of ID within children with CP [57]. For a more accurate assessment of intellectual levels in children with CP (and particularly for children with more severe motor impairment), cognitive tests should have greater adaptation with consideration of motor functioning [57].

Behavioral concerns appear to be common in children with CP, and the presence of early behavioral concerns (including ADHD and oppositional defiant disorder) is associated with later emotional disorders [14]. Previous reports of behavior difficulties in children with CP have been high, with 39.4% of parents reporting behavioral concerns in the borderline to abnormal range [58]. Behavioral concerns in children with CP are often associated with other factors or comorbidities, such as ID, sleep problems, and ASD symptoms [20], difficulties with friendships and bully victimization [25], and pain [11]. While parenting intervention programs have shown promise for behavioral concerns in children with CP [10], interventions focused on associated characteristics may also be helpful in reducing behavioral concerns.

Research over the past five years indicates that children and adolescents with CP have higher odds of anxiety and depression [12]. Previous research has reported prevalence rates of 33% for depression and 31% for anxiety in a sample of adolescents and young adults with CP [7]. However, associated characteristics such as pain, sleep, and fatigue may contribute to higher reported rates of these diagnoses [32]. The prevalence of anxiety has been shown to be higher in children with chronic medical conditions [59], which may also be the case for children with CP.

### 4.2. Academic Functioning

The studies focusing on academic and learning concerns for children with CP over the last five years highlight the significant academic and cognitive challenges faced by children with CP and underscore the complex interaction of neurological, cognitive, and educational factors impacting this population. The presence of learning disorders may be high in this population, with over half exhibiting at least one learning disorder and close to 40% having more than one learning disorder [38]. This is consistent with previous reports of learning disorders in this population, as Frampton et al. [60] were surprised to find that 36% of their sample (149 children with hemiplegia from London with CP) had specific learning disorders. Specific cognitive deficits, particularly in working memory and visual-spatial processing, may shape the outcomes for children with CP, indicating potential areas for targeted cognitive interventions [35]. The contrasting findings between Pereira et al. [39] and Wotherspoon et al. [40] imply that interventions focused on academic functioning and engagement must be carefully tailored to meet the cognitive and physical needs of children with CP to ensure active participation and positive outcomes. The academic differences between children with CP and typically developing peers [36,37] underscores the need for academic support that provides children with CP an equitable chance for academic success, especially given their differences to children with SLDs. These findings are consistent with previous literature that suggests that children with CP should receive specific academic interventions [9].

Two different authors argued that learning disorders in children with CP should be classified as “non-specific” and secondary due to impairments in global cognitive functions that exist in children with CP [36,38]. These findings suggest that educational difficulties in CP are often interwoven with broader cognitive deficits, complicating traditional classifications of specific learning disorders. The generalizability of the various findings is also complicated due to the small numbers of participants and the exclusionary criteria that existed. By trying to reduce confounding variables and excluding children with CP who have comorbid neurodevelopmental disorders, the authors of the various studies likely increased selection bias which decreases generalizability. However, these findings have implications for youth with CP within educational settings. Should learning difficulties be considered “non-specific” or secondary to global cognitive functioning impairment, it highlights the need for formalized, neuropsychological assessment of all children with CP, not just those with identified learning disorders. Direct intervention to improve these specific cognitive or executive functions may benefit children with CP throughout their educational career. Future research is undoubtedly necessary to further understand the multidimensional nature of educational challenges in children with CP, as well as to develop educational support to best address the diverse learning needs of children with CP.

### 4.3. Interventions

The interventions reviewed illustrate a recent emphasis on attention to critical aspects of overall child development. Given that children with CP often endure challenges secondary to their primary diagnosis, therapeutic interventions should aim to address multiple areas of functional impairment. Future research should continue to identify interventions that improve multiple functional domains.

There are methodological concerns regarding intervention studies included in this review. Of the studies included in our intervention section, eight were true intervention studies [41,42,43,45,46,47,48] and one was an assessment follow-up of a previous randomized controlled trial [44]. The additional two studies provided information relevant to interventions, and, as such, were included in this section of the review [12,22]. Mixed results were found across these different interventions. Of the seven active intervention studies, all used randomization for allocation to groups [41,42,43,45,46,47,48]. Control groups were primarily no intervention or treatment as usual [41,42,43,45,47], and two studies utilized waitlist control groups [46,48]. Stronger conclusions could be drawn against a stronger, more active control group. Sample sizes in all intervention studies were small, and no studies included children over the age of twelve, limiting generalization to clinical populations. Outcome measures utilized were frequently self-reported, and there was a limited overlap in outcome measures, as the studies included often focused on different targets or outcomes.

Five studies reported information about participants’ motor function ability [42,43,44,45,47]. Of these, only two studies included participants with severe mobility impairment (GMFCS Level V) [42,47]. Children with severe mobility restrictions may have unique psychological and social needs that are not adequately addressed by interventions targeting children with higher mobility. There should be a significant push towards intervention research focused on children with the most severe mobility impairments, particularly, interventions that are either adapted for this population or interventions explicitly tailored address this group’s needs. The lack of consistent reporting of mobility functioning in survey samples, combined with the tendency to only include children with greater mobility, raises concerns about the generalizability of intervention outcomes. This gap in the literature highlights the need for more inclusive research that accounts for the full spectrum of CP-related impairments.

Three studies reviewed included interventions and outcome measures specifically addressing attentional deficits in children with CP [12,41,43]. Given the rates of ADHD within the CP population, there is a need for interventions focused on attention. Behavioral concerns or aggression were also focused on by interventions reviewed in this paper [42,45,47]. A previous RCT found that an evidence-based parenting intervention (Stepping Stone Triple P) and Acceptance and Commitment Therapy delivered to parents of children with CP were associated with a decrease in child behavioral problems and changes in parent behavior [10] and could serve as a strong control for these novel interventions.

One interesting area of future research would be further focus on cognitive behavioral approaches for the CP population. In a scoping review on Cognitive Behavioral therapy (CBT) for individuals with CP, Silberg et al. [61] reported that CBT can be adapted for the CP population. The literature base on CBT for this group is limited by small sample sizes and a wide age range within the available studies [61]. Positive outcomes were reported on various objectives in all studies in a scoping review on this subject, but no concrete determination on the evidence of CBT for individuals with CP could be drawn [61].

With consideration of mixed results of intervention studies in this review and the wide range of targets addressed by interventions, we feel that there is a current gap in the literature on evidence-based psychological interventions for the pediatric CP population. Research efforts should be made to evaluate psychological interventions with strong research evidence for typically developing populations or children with neurodevelopmental disabilities for children and adolescents with CP.

### 4.4. Assessment Concerns

The concern for appropriate assessment and screening tools or approaches is not limited to the assessment of intellectual functioning for children with CP. There is a pressing need for assessment tools specifically designed for children with CP, especially those with severe cognitive and communication limitations. Overall, diagnostic difficulties may be observed for children with CP who have more associated impairments [19], and associated functional or clinical impairment is common for children with CP [2]. The severity of symptoms, including ID and GMFCS level V, inhibiting the ability to appropriately assess or diagnosis children with CP has been documented before [4]. Regular screening for behavioral issues is essential, but existing tools are often unsuitable for children with more severe disabilities; thus, research is needed to develop appropriate assessment methods [30]. With consideration of reported rates of comorbid diagnoses and symptoms, children with CP should be screened for both ASD and ADHD [6]. Regarding cognitive impairment, it is recommended to perform regular global assessment in children with an early CP diagnosis from early ages until late childhood [9]. Given the trajectories of mental health diagnoses within this population [14,15], screening for mental health concerns across childhood is warranted. One screening measure that was seen frequently across this review, and may be helpful in assessing, is the Strengths and Difficulties Questionnaire, which was found to be satisfactory for this population at pre-adolescence [15].

### 4.5. Limitations

There are limitations to the current review. While we utilized a search strategy with clear inclusion and exclusion criteria, narrative reviews may be more susceptible to bias in selection of articles. The first author, a doctoral-level psychologist, screened all abstracts and utilized the inclusion and exclusion criteria to ultimately select articles included in this review. We acknowledge that this author’s professional and personal perspective and experiences may have influenced this selection process. Strict adherence to the inclusion and exclusion criteria was used to limit this bias. We excluded studies not written in English, which may have contributed to bias and limited information reviewed to not include the full scope of available evidence. We chose to include systematic reviews that only included a meta-analysis, as our aim was to review the most recent research literature published over the past five years. This choice may have led to the omission of novel perspectives or insights gained from the synthesis of available literature.

### 4.6. Implications for Clinical and Academic Practice:

- Children and adolescents with CP should be screened regularly for mental health concerns and disorders, starting as early as the preschool years. This is particularly true for symptoms consistent with ADHD, ASD, and behavioral concerns.
- Considering that early symptoms of mental health are predictive of later mental health concerns and that symptoms are likely to persist, early intervention for mental health concerns should be provided to children and adolescents in addition to preventative care or addressing subclinical mental health symptoms.
- Parenting behavioral interventions should be recommended to families of children with behavioral concerns.
- Caution should be taken when choosing mental and cognitive assessment tools and results interpreted with caution, particularly for children with more severe motor impairments. The Strengths and Difficulties Questionnaire may be a useful screening tool for use with youth with CP.
- Children with CP should be assessed regularly for global and specific learning concerns within academic settings to allow for identification of needs and tailored academic intervention.

## 5. Conclusions

The current paper has expanded on the last review focused on the prevalence of mental health concerns in the pediatric CP population published in 2018 [3] to include a significantly larger body of research evidence with a wider range of mental health diagnoses or concerns, academic functioning, and interventions. This paper adds to the literature an extensive review of recent research on this subject and the identification of research gaps to better understand the best ways to support children with CP. Youth with CP are at increased risk for mental and behavioral symptoms and diagnoses, and this increased risk spans diagnostic categories. Children with CP commonly experience academic concerns or impaired academic functioning and should be assessed for these concerns across childhood. Appropriate assessment of children with CP, either psychological, functional, or intellectual, continues to be a significant concern (particularly for children with greater motor or cognitive impairment), and there is a need for tailored assessment approaches. Intervention studies have examined both parent- and child-focused interventions and spanned many areas of functioning; however, results are mixed, and evaluation of established psychological interventions should be conducted for this population.

## Data Availability

No new data were created or analyzed in this study. Data sharing is not applicable to this article.

## Abbreviations

The following abbreviations are used in this manuscript:

CP	Cerebral Palsy
ADHD	Attention-Deficit/Hyperactivity Disorder
ASD	Autism Spectrum Disorder
ID	Intellectual Disability
GMFCS	Gross Motor Function Classification System
EAA	Equine-Assisted Activities
PACT	Parenting Acceptance and Commitment Therapy

## Appendix A. Search Strings

The three searches on PubMed, focused on MeSH/title searches, included:

Cerebral palsy and mental health

(“Cerebral Palsy”[Mesh] OR “cerebral palsy”[ti] OR “early brain lesion”[ti] OR “perinatal stroke”[ti] OR “hemiplegia”[ti] OR “diplegic”[ti] OR “dyskinetic cerebral palsy”[ti]) AND (“Child”[Mesh] OR “Adolescent”[Mesh] OR child*[ti] OR adolescent*[ti]) AND (“Behavioral Symptoms”[Mesh] OR “Stress, Psychological”[Mesh] OR “Mental Health”[Mesh] OR “Mental Disorders”[Mesh] OR behavioral[ti] OR psychological[ti] OR emotional[ti] OR “mental health”[ti] OR “mental disorder*”[ti] OR psychiatric[ti])

Cerebral Palsy and Interventions

(“Cerebral Palsy”[Mesh] OR “cerebral palsy”[ti] OR “early brain lesion”[ti] OR “perinatal stroke”[ti] OR “hemiplegia”[ti] OR “diplegic”[ti] OR “dyskinetic cerebral palsy”[ti]) AND (“Child”[Mesh] OR “Adolescent”[Mesh] OR child*[ti] OR adolescent*[ti]) AND (“Psychotherapy”[Mesh] OR “Psychosocial Intervention”[Mesh] OR “psychological intervention*”[ti] OR “psychosocial intervention*”[ti] OR “Behavior Therapy”[Mesh] OR “behavior therap*”[ti] OR “behavioral intervention*”[ti])

Cerebral Palsy and Academic Performance/Learning Disorders

(“Cerebral Palsy”[Mesh] OR “cerebral palsy”[ti] OR “early brain lesion”[ti] OR “perinatal stroke”[ti] OR “hemiplegia”[ti] OR “diplegic”[ti] OR “dyskinetic cerebral palsy”[ti]) AND (“Child”[Mesh] OR “Adolescent”[Mesh] OR child*[ti] OR adolescent*[ti]) AND (“Academic Performance”[Mesh] OR “academic performance”[ti] OR “academic success”[ti] OR academic[ti] OR “Learning Disabilities”[Mesh] OR “Specific Learning Disorder”[Mesh] OR “learning disabilit*”[ti] OR “learning disorder*”[ti] OR “specific learning disorder*”[ti])

The three searches on Scopus, focused on titles/abstracts, included the following:

Cerebral Palsy and Mental Health

(“cerebral palsy” or “early brain lesion” or “perinatal stroke” or “hemiplegia” or “diplegic” or “dyskinetic cerebral palsy”) AND (child* OR adolescent) AND (behavioral OR psychological OR emotional OR “mental health” OR “mental disorder*” OR psychiatric)

Cerebral Palsy and Interventions

(“cerebral palsy” or “early brain lesion” or “perinatal stroke” or “hemiplegia” or “diplegic” or “dyskinetic cerebral palsy”) AND (child* OR adolescent) AND (“academic performance” OR “academic success” OR academic OR “learning disabilit*” OR “learning disorder*” OR “specific learning disorder*”)

Cerebral Palsy and Academic Performance/Learning Disorders

(“cerebral palsy” or “early brain lesion” or “perinatal stroke” or “hemiplegia” or “diplegic” or “dyskinetic cerebral palsy”) AND (child* OR adolescent) AND (“psychological intervention*” OR “psychosocial intervention*” OR “behavior therap*” OR “behavioral intervention*”)

## Appendix B

**Table A1:** Summary of purpose, design, sample, and relevant results of studies included in review.

Author/Year	Purpose	Study Design	Sample	Relevant Findings
Horwood L, Li P, Mok E, Oskoui M, Shevell M, Constantin E (2019) [34]	Determine the prevalence of behavioral challenges in preschool and school-aged children with CP and assess the association between behavioral challenges and sleep problems, nighttime pain, and child characteristics.	Cross-sectional study	Caregivers of 113 children with CP	Approximately 26% of children with CP had behavioral challenges. Sleep problems and nighttime pain were also associated with behavioral challenges. Sleep and behavioral problems were highly associated even when adjusting for nighttime pain and age.
Whitney DG, Warschausky SA, Peterson MD. (2019) [11]	Examine the prevalence of mental health disorders among children with and without CP. Examine how physical risk factors in children with CP may mitigate any elevated risk of mental disorders in the population.	Cross-sectional study	111 children with and without CP	Children with CP had higher odds of developing mental health disorder, with the exception of ADHD. Risk for depression decreased when adjusting for physical factors, but anxiety and behavioral concerns remained high. When assessed individually, risk for depression was no longer increased when controlling for low physical activity and pain.
Whitney DG, Peterson MD, Warschausky SA. (2019) [25]	Examine how social factors may mitigate elevated risks of mental health disorders in children with CP.	Cross-sectional study	111 children with and without CP	Children with CP had a higher likelihood of developing anxiety and behavioral problems after controlling for sociodemographic factors and chronic pain. This risk remained high even after adjusting for being engaged in activities. However, when adjusting for challenges with friendships, the risk for anxiety was no longer increased in children with CP. After adjusting for bullying, the odds of behavioral problems were attenuated but anxiety risk remained high.
Påhlman M, Gillberg C, Himmelmann K. (2019) [18]	Describe associated impairments and motor function in children with CP. Examine the association between CP type, motor functioning, and associated impairments in a population group. Compare associated impairment in the same children at school age.	Population-based study	264 children with CP born between 1999 and 2006 identified through the CP Register of western Sweden (limited to the county of Vastra Gotaland)	Associated impairments were found in 75% of this sample. ID was present in 53%, ASD was present in 18%, and ADHD was present in 21%. Except for ADHD and ASD, all impairments increased with more severe motor impairment.
Shahriari, Y., Ghasemzadeh, S., & Vakili, S. (2019) [47]	Examine how effective Child Centered Play Therapy is in improving internalizing and externalizing behaviors for children with CP.	Applied and quasi-experimental with pre- and post-tests	30 participants with CP and comorbid medical diagnosis of paraplegia and mild to moderate monoplegia, randomly assigned evenly (n = 15) between control and intervention groups	A significant reduction in anxiety, depression, offense, and aggression was observed in the experimental group at both post-test and follow-up, as compared to the control group. There was no change in externalizing offensive behavior.
Pereira A, Rosário P, Lopes S, Moreira T, Magalhães P, Núñez JC, Vallejo G, Sampaio A. (2019) [39]	Examine the efficacy of an educational program focused on the promotion of school engagement in children with CP.	Quasi-experimental design (no control group)	15 children with CP recruited from CP rehab centers in Portugal	Participation in the program improved behavioral engagement, emotional engagement, and cognitive engagement (the dimensions of school engagement).
Gardiner E, Miller AR, Lach LM (2020) [33]	Examined the extent to which behavioral problems represent a functional characteristic that crosses diagnostic boundaries.	Cross-sectional study	179 caregivers of children who have CP, ASD, and global developmental disability/ID	All three groups had matching profile topographies, indicating substantial difficulties with hyperactivity/inattention.
Rackauskaite G, Bilenberg N, Uldall P, Bech BH, Østergaard J (2020) [13]	Compare prevalence of mental disorders in a sample of children and adolescents with CP and age/sex matched controls. Examine the association between motor function and mental disorders in youth with CP.	Register-linkage follow-up study	977 children with CP born between 1997 and 2003 identified from the Danish National Cerebral Palsy Registry and 2627 children without CP matched by age and gender	Prevalence of mental disorders was significantly higher in children with CP compared to controls. ID was associated with motor function.
McMahon J, Harvey A, Reid SM, May T, Antolovich G. (2020) [30]	Describe the prevalence of and factors associated with anxiety in children with CP and determine how often providers screen for and manage anxiety in children with CP.	Cross-sectional observational study	172 families of children with CP participated	A total of 38% of children were found to have clinically significant anxiety based on parent report, and 46% had clinically significant anxiety on child report. Girls were twice as likely to have anxiety. Parent and child reports were highly correlated. Based on reports on the Screen for Child Anxiety Related Disorders measure, 16 children (43%) with clinically significant anxiety were not identified by a provider.
Påhlman M, Gillberg C, Wentz E, Himmelmann K. (2020) [19]	Estimate the screen-positive rates of ADHD and ASD in a group of children with CP.	Screening study with parent-completed questionnaires	264 school-aged children with CP identified through the CP registrar of western Sweden	One-third screened positive for ASD and half screened positive for ADHD. Children with epilepsy, impaired speech, and ID screened positive for ADHD and ASD more often. Severe motor impairment was associated with screening positive for ASD.
Leader G, Molina Bonilla P, Naughton K, Maher L, Casburn M, Arndt S, Mannion A (2021) [20]	Identify frequency of GI symptoms, sleep problems, internalizing/externalizing symptoms, and ASD symptoms in a sample of children with CP. Examine impact of comorbidities on frequency and severity of behavioral problems in this sample.	Parent-completed questionnaires	104 youth with CP	High frequency of behavior problems, sleep problems, gastrointestinal symptoms, ASD symptoms, and internalizing/externalizing symptoms were found in this sample. Relationships were found between the following: sleep problems and behavior problems; GI symptoms and sleep problems; GI symptoms and internalizing/externalizing problems. Behavioral problems were predicted by sleep problems, internalizing/externalizing symptoms, ID, and ASD symptoms.
Salie R, Eken MM, Donald KA, Fieggen AG, Langerak NG (2021) [31]	Investigate the prevalence and level of disability due to pain, health-related quality of life, and mental health in adolescents and adults with CP.	Case-control study	31 adolescents and 30 adults with CP and typically developing peers	Adolescents and adults with CP reported experiencing more frequent pain in their lower limbs with associated higher levels of disability due to the pain, lower health-related quality of life compared to typically developing peers. There were no differences found in mental health outcomes for those with CP compared to typically developing peers.
Bjorgaas HM, Elgen IB, Hysing M. (2021) [14]	Examine trajectories of mental health disorders and association with risk factors in children with CP.	Assessment at the age 7 and 11	47 children with CP born 2001–2003 living in the Western Health Region of Norway	Significant increase in prevalence of emotional disorders between ages of 7 and 11. Stable prevalence of behavioral disorders. Half of the sample met criteria for a mental health disorder at both time points. Subthreshold mental health disorders at age 7 predicted mental health disorders at age 11.
Påhlman M, Gillberg C, Himmelmann K. (2021) [17]	Estimate the prevalence of ASD and ADHD in a population of school aged children with CP. Describe diagnoses in relation to associated characteristics. Compare screening rates to diagnoses.	Screening and assessments	200 children with CP	A total of 45% of children were diagnosed with ASD, ADHD, or both. ASD was predicted by ID and ADHD. ADHD was predicted by ID and ASD.
Cummins D, Kerr C, McConnell K, Perra O. (2021) [27]	Describe the association between spastic CP subtypes and severe ID across gestational age categories, standardized birthweight scores, motor severity of CP, and participants’ sex. Investigate how gestational age categories moderate the association between spastic CP subtypes and severe ID, while controlling for standardized birthweight scores and participant sex.	Population-based, cross-sectional study using data from the Northern Ireland Cerebral Palsy Register (NICPR)	1452 individuals with prenatal or perinatal spastic CP, data from the Northern Ireland Cerebral Palsy Register (NICPR)	A total of 40.4% of children were diagnosed with ID. Severe ID was more likely in infants born at term compared to those born before 36 weeks. Infants smaller than their gestational age were more at risk of having severe ID compared to those of a normative birth weight.
Casseus M, Cheng J. (2021) [22]	Identify the proportion of children with CP who have an unmet need for care coordination. Examine the physical and functional health of this population. Examine if unmet need differs by CP severity or demographic characteristics.	Survey Analysis	102,341 NSCH respondents from the years of 2016, 2017, and 2018	Children diagnosed with CP had significantly higher prevalence of all comorbid conditions and functional disabilities. More than half of children with CP had an unmet need for care coordination.
Ahn B, Joung YS, Kwon JY, Lee DI, Oh S, Kim BU, Cha JY, Kim JH, Lee JY, Shin HY, Seo YS. (2021) [41]	Examine the effectiveness of equine-assisted activities on attention and quality of life in children with CP. Examine comorbidity between CP and ADHD and the effectiveness of this intervention for children with this comorbidity.		46 children diagnosed with CP	Children with CP in the excursive group demonstrated improvement in attention. Sub-analysis of children with comorbid CP and ADHD demonstrated improvement in attention and perceived social skills after 16 of equine-assisted activities, compared to the control group.
Jarl J, Alriksson-Schmidt A (2021) [37]	Investigate whether adolescents with CP in Sweden have lower school achievement and if school achievement varies by disability-specific factors	Population-based register study	Children with CP in Sweden, born between 1990 and 1999 with a matched comparison group from the general population; for compulsory school, 1648 cases with CP and 16,838 comparators; for secondary school, (voluntary in Sweden) 2541 cases with CP and 122,999 comparators	Adolescents with CP had a substantial reduction in school outcomes (partly explained by the high presence of ID). Children with CP had lower levels of education when compared to the general population, even after controlling for ID and motor difficulties.
de Freitas Feldberg SC, da Silva Gusmão Cardoso T, Santos FH, Muszkat M, Bueno OFA, Berlim de Mello C. (2021) [36]	Investigate aspects of numerical cognition in a sample of bilateral and unilateral CP and whether the cognitive deficits and learning difficulties follow the pattern of primary or secondary dyscalculia.	Exploratory cross-sectional study	19 children/adolescents recruited from a multidisciplinary center for neurodevelopmental disorders in Sao Paulo, Brazil compared to 31 typically developing children	The unilateral CP group showed significantly lower performance on the writing and reading subtests compared to the traditionally developing group. Both the unilateral and bilateral CP groups had significantly lower arithmetic performance than the traditionally developing group. CP groups had worse performance on tasks of working memory and cognition as well as worse performance on tests involving numerical cognition. Concluded that the mathematics learning disorder was secondary to the neurological disorder and that the impairment in math skills was likely due to global cognitive dysfunctions (i.e., visual-spatial processes and working memory).
De Clercq LE, Soenens B, Dieleman LM, Prinzie P, Van der Kaap-Deeder J, Beyers W, De Pauw SSW (2022) [23]	Address the joint contribution of parent-related parenting behaviors and child personality on psychosocial outcomes in children with CP.	Longitudinal study	118 families of children with CP	Externally controlling and autonomy-supportive parenting behavior remained stable over time. Externally controlling parenting related to increased behavioral problems was found in children who were lower on extraversion, conscientiousness, and imagination. Autonomy-supportive parenting was related to higher levels of psychosocial strengths and was found mostly in children who were higher on emotional stability.
Bjorgaas HM, Elgen IB, Hysing M (2022) [15]	Assessed changes in parent-rated mental health problems and differences in mental health problems according to informants. Assessed the validity of the Strengths and Difficulties Questionnaire for psychiatric disorders.	Survey analysis	67 children with CP from school-starting age to pre-adolescence	Parental scores on the SDQ were elevated on emotional, hyperactivity, and total number of problems. Self-reports of impact of mental health were lower than parent reports. Parents and pre-adolescents reported elevated impact of mental health compared to teacher reports, especially on mood, conduct, and total problems. Strengths and Difficulties Questionnaire was found to be satisfactory in screening for mental health concerns in youth with CP.
Laporta-Hoyos, O., Pannek, K., Pagnozzi, A. M., Whittingham, K., Wotherspoon, J., Benfer, K., Fiori, S., Ware, R. S., & Boyd, R. N. (2022) [35]	Determine which combination of clinical scores is associated with psychological outcomes (cognitive, academic, and executive functioning as well as psychological adjustment) in children and adolescents with spastic motor type CP.	Cross-sectional analysis of data	101 children and adolescents with CP recruited from the Queensland Cerebral Palsy and Rehabilitation Research Centre	Lesions in the medial dorsal thalamus and parietal lobe lesions significantly accounted for poorer verbal proxy IQ ratings. Compared to those with unilateral left-sided lesions, those with bilateral lesions performed better in verbal cognitive functions. Children with CP with ventral posterior lateral thalamus lesions had better parent-related behavioral displays of executive functioning when controlling for the presence of an epilepsy diagnosis. The semi-quantitative scale for structural MRI scoring has implications for personalized interventions for children with CP.
PÅhlman M, Gillberg C, Himmelmann K. (2022) [16]	Describe and compare neuroimaging patterns in a group of children with CP and relate findings to ADHD diagnosis, ASD diagnosis, and other associated characteristics.	Population-based study	184 children with CP born between 1999 and 2006 identified through the CP register of western Sweden	ASD and ADHD were common in all neuroimaging patterns. The highest prevalence of ASD was found in children with predominant white matter injury. The highest prevalence of ADHD was found in children with middle cerebral artery infarction.
Mak C, Whittingham K, Cunnington R, Chatfield M, Boyd RN. (2022) [44]	Examine treatment effects at a 6-month follow-up and retention of a RCT of mindfulness-based yoga program (MiYoga) for CP.	6-month follow-up of a waitlist control randomized controlled trial	41 parent-child dyads with a child diagnosed with CP participated in initial intervention; 23 of these dyads completed the 6-month follow-up	Child executive and physical function, in addition to parent well-being, improved significantly from pre-intervention to 6-month follow-up, possibly demonstrating a delayed effect of the intervention. No significant changes between pre-intervention or post-intervention to 6-month follow-up on child attention variables or parental mindfulness.
Leader G, Mooney A, Chen JL, Whelan S, Naughton K, Maher L, Mannion A. (2022) [21]	Examined the frequency of comorbidities in children with ASD alone, CP alone, and those with comorbid CP and ASD.	Screening study	96 children with a diagnosis of CP, ASD, or comorbid CP and ASD	Significant group differences in sleep problems, social communication, and adaptive behavior. ID significantly predicted levels of adaptive behavior.
Olusanya BO, Gladstone M, Wright SM, Hadders-Algra M, Boo NY… (2022) [28]	Report the best available evidence on the global and regional prevalence of CP and developmental ID and the associated “years lived with disability” among children under five years of age in 2019.	Cross-sectional analysis of data	GBD-WHO Rehabilitation Database-data from 204 countries	CP has the highest prevalence in low- and middle-income countries. Of the 662.8 million children under five, 8.1 million (or 1.2%) were estimated to have CP and 16.11 million (or 2.4%) were estimated to have ID.
Ödek, Uğur & Özcan, kürşat & Ozyurt, Gonca & Akpinar, Selcuk. (2022) [45]	Examine the benefits of equine-facilitated activities on symptoms of anxiety, aggression, empathy, and emotional regulation in children with CP. Examine the impact of equine facilitated activities on maternal depression and anxiety.	Between-groups design	20 children with CP and their mothers	Eight weeks of adaptive riding was associated with decreases in the children’s anxiety and aggression, in addition to decreased maternal depression. The intervention was associated with improvements in emotional regulation and empathy.
Whittingham K, Sheffield J, Mak C, Wright A, Boyd RN. (2022) [48]	Examine the effectiveness of Parenting Acceptance and Commitment Therapy in improving the emotional availability of the parent/child relationship, parental psych well-being, child psych adjustment, and quality of life.	RCT with waitlist control	67 parents of children with CP	Two components of parental emotional availability (parental non-intrusiveness and child involvement) significantly improved with intervention. Parents reported improved comfort with their child’s CP diagnosis, higher likelihood to seek help, higher likelihood to connect with others, and greater meaningful living.
Samijn B, Van den Broeck C, Plasschaert F, Pascal A, Deschepper E, Hoebeke P, Van Laecke E. (2022) [46]	Examine the effectiveness of incontinence training with urotherapy in a sample of children with CP.	Populated-based case-control study	21 children with CP and 24 typically developing children with either daytime-only or both daytime and nocturnal enuresis	For children with CP, the effectiveness rate of incontinence training is lower. Compared to typically developing peers, changes from incontinence training are slower to occur in children with CP.
Cribb CF, Keko M, Creveling S, Rochani HD, Modlesky CM, Colquitt G (2023) [12]	Determine the relationship between prevalence of mental health disorders and participation in physical activity and sports.	Survey analysis	458 children with CP and 40,091 typically developing children whose parents participated in the 2016–2020 NSCH	Children with CP had a higher prevalence of mental health disorders and were more likely to receive mental health care. Children with CP had higher rates of anxiety, depression, behavioral disorders, and ADHD. Participation in sports and daily physical activity was associated with reduced likelihood of some conditions.
Alibakhshi, H., Simin ghalamaval, M., Ayoubi Avaz, K., Salmani, M., Pahlavanian, A., Motaharinezhad, F., Kanani, Z (2023) [42]	Examine the effectiveness of teaching communication skills to mothers of children with CP and evaluate child behavioral problems.	Semi-experimental design with pre- and post-test assessment	42 mothers of children with CP who were displaying behavioral problems	Communication skills group demonstrated significant reduction in the following areas of measurement: aggression & hyperactivity, social incompatibility, antisocial behaviors, anxiety and depression, and general behavioral problems.
Testani D, McMorris CA, Clark CA, Sanguino H, Condliffe EG, Noel ME, Kopala Sibley DC, Brunton LK (2024) [32]	Examine individual and cumulative impacts of physiological symptoms on anxiety and depression in youth with CP.	Cross-sectional observational study	40 youth with CP and their caregivers	Youth with CP experienced elevated levels of physiological symptoms and elevated anxiety and depression symptoms. Factors also contributing to caregiver reports of the child’s anxiety and depression included fatigue, severe pain, sleep efficiency, and level of physical activity.
Fitneva SA, Corbett BA, Prasad AN. (2024) [29]	Analyze behavioral outcomes who had severe epilepsy, CP, and ID compared to typically developing peers.	Review and analysis of longitudinal survey	10,879 2–3-year-old children from 3 cycles of Canada’s National Longitudinal Survey of Children and Youth	Children with neurodevelopmental disabilities and significant differences in behavioral outcomes at younger ages (higher levels of hyperactivity/inattention, challenges with prosocial behaviors, separation anxiety, and mood/anxiety concerns) when compared to typically developing peers.
Chen Q, Chen M, Bao W, Strathearn L, Zang X, Meng L, Xu G. (2024) [24]	Examine associations between CP with ADHD and ASD.	Large-scale nationwide population-based study	177,899 children between the ages of 3 and 17 years old. Data taken from the National Health Interview Survey from 1997–2003 and 2008–2018	Children with CP had a higher prevalence of ADHD and ASD.
Casseus M, Cheng J, Reichman NE. (2024) [26]	Estimate associations between clinical and functional characteristics and a diagnosis of ADHD in a sample of children and young adults with CP.	Retrospective, cross-sectional studying using electronic health records	1145 children and young adults with CP	A total of 18.1% of the sample had a diagnosis of ADHD. Those with bilateral spastic CP had lower odds of ADHD. GMFCS levels III-IV were associated with a lower odds of ADHD.
Chen YC, Chang WP, Liang KJ, Chen CL, Chen HY, Chen SP, Chan PS (2024) [43]	Examine the effects of neurofeedback training on attention task performance in children with CP and attention deficits.	Two-arm parallel design randomized trial	19 children with both CP and attention deficits	The neurofeedback intervention group demonstrated decreased theta/beta ratios at post-training compared to the control group and within-group improvement during the training intervention. Neurofeedback group demonstrated a trend for decreased omission rates on an assessment of attention related problems.
Micheletti S, Galli J, Vezzoli M, Scaglioni V, Agostini S, Calza S, Merabet LB, Fazzi E. (2024) [38]	Investigate the prevalence and clinical manifestations of learning disorders (reading, writing, and mathematics disorders) in a group of children with CP with normal verbal IQ and reduced motor involvement; explore how the clinical profile from a group of children with CP and learning disorders differs from that of children with CP without learning disorders and children with SLDs.	Prospective cross-sectional study	42 children with CP and 60 children with SLDs	A total of 59% of the children with CP had reading, writing, or mathematics disorders. Children with CP with learning disorders had a low performance IQ, normal phonological awareness, and working memory difficulties. Children with SLDs had normal performance IQ, impaired phonological awareness, and mild working memory difficulties.
Wotherspoon J, Whittingham K, Sheffield J, Boyd RN. (2024) [40]	Test the efficacy of a novel web-based cognitive rehabilitation program (Strengthening Mental Abilities through Relational Training) for children between 8 and 12 years old with mild to moderate congenital CP.	Mixed methods sequential explanatory design	21 Australian children with CP	No group differences found between the experimental group and the control group (perhaps due to specific challenges in engaging with and completing the training).

## References

[1] Graham H.K., Rosenbaum P., Paneth N., Dan B., Lin J.P., Damiano D.L., Becher J.G., Gaebler-Spira D., Colver A., Reddihough D.S. (2016). Cerebral Palsy. Nat. Rev. Dis. Primers.

[2] Novak I., Hines M., Goldsmith S., Barclay R. (2012). Clinical Prognostic Messages from a Systematic Review on Cerebral Palsy. Pediatrics.

[3] Downs J., Blackmore A.M., Epstein A., Skoss R., Langdon K., Jacoby P., Whitehouse A.J.O., Leonard H., Rowe P.W., Glasson E.J. (2018). The Prevalence of Mental Health Disorders and Symptoms in Children and Adolescents with Cerebral Palsy: A Systematic Review and Meta-Analysis. Dev. Med. Child Neurol..

[4] Bjorgaas H.M., Hysing M., Elgen I. (2012). Psychiatric Disorders among Children with Cerebral Palsy at School Starting Age. Res. Dev. Disabil..

[5] Parkes J., White-Koning M., Dickinson H.O., Thyen U., Arnaud C., Beckung E., Fauconnier J., Marcelli M., McManus V., Michelsen S.I. (2008). Psychological Problems in Children with Cerebral Palsy: A Cross-Sectional European Study. J. Child Psychol. Psychiatry.

[6] Craig F., Savino R., Trabacca A. (2019). A Systematic Review of Comorbidity between Cerebral Palsy, Autism Spectrum Disorders and Attention Deficit Hyperactivity Disorder. Eur. J. Paediatr. Neurol..

[7] Gorter J.W., Fehlings D., Ferro M.A., Gonzalez A., Green A.D., Hopmans S.N., McCauley D., Palisano R.J., Rosenbaum P., Speller B. (2022). Correlates of Mental Health in Adolescents and Young Adults with Cerebral Palsy: A Cross-Sectional Analysis of the MyStory Project. J. Clin. Med..

[8] Sigurdardottir S., Indredavik M.S., Eiriksdottir A., Einarsdottir K., Gudmundsson H.S., Vik T. (2010). Behavioural and Emotional Symptoms of Preschool Children with Cerebral Palsy: A Population-Based Study. Dev. Med. Child Neurol..

[9] Fluss J., Lidzba K. (2020). Cognitive and Academic Profiles in Children with Cerebral Palsy: A Narrative Review. Ann. Phys. Rehabil. Med..

[10] Whittingham K., Sanders M., McKinlay L., Boyd R.N. (2014). Interventions to Reduce Behavioral Problems in Children with Cerebral Palsy: An RCT. Pediatrics.

[11] Whitney D.G., Warschausky S.A., Peterson M.D. (2019). Mental Health Disorders and Physical Risk Factors in Children with Cerebral Palsy: A Cross-Sectional Study. Dev. Med. Child Neurol..

[12] Cribb C.F., Keko M., Creveling S., Rochani H.D., Modlesky C.M., Colquitt G. (2023). Mental Health, Physical Activity, and Sports among Children with Cerebral Palsy. Child Care Health Dev..

[13] Rackauskaite G., Bilenberg N., Uldall P., Bech B.H., Østergaard J. (2020). Prevalence of Mental Disorders in Children and Adolescents with Cerebral Palsy: Danish Nationwide Follow-up Study. Eur. J. Paediatr. Neurol..

[14] Bjorgaas H.M., Elgen I.B., Hysing M. (2021). Trajectories of Psychiatric Disorders in a Cohort of Children with Cerebral Palsy across Four Years. Disabil. Health J..

[15] Bjorgaas H.M., Elgen I.B., Hysing M. (2022). Mental Health in Pre-Adolescents with Cerebral Palsy: Exploring the Strengths and Difficulties Questionnaire as a Screening Tool in a Follow-Up Study Including Multi-Informants. Children.

[16] PÅhlman M., Gillberg C., Himmelmann K. (2022). Neuroimaging Findings in Children with Cerebral Palsy with Autism and/or Attention-Deficit/Hyperactivity Disorder: A Population-Based Study. Dev. Med. Child Neurol..

[17] Påhlman M., Gillberg C., Himmelmann K. (2021). Autism and Attention-Deficit/Hyperactivity Disorder in Children with Cerebral Palsy: High Prevalence Rates in a Population-Based Study. Dev. Med. Child Neurol..

[18] Påhlman M., Gillberg C., Himmelmann K. (2019). One-Third of School-Aged Children with Cerebral Palsy Have Neuropsychiatric Impairments in a Population-Based Study. Acta Paediatr..

[19] Påhlman M., Gillberg C., Wentz E., Himmelmann K. (2020). Autism Spectrum Disorder and Attention-Deficit/Hyperactivity Disorder in Children with Cerebral Palsy: Results from Screening in a Population-Based Group. Eur. Child Adolesc. Psychiatry.

[20] Leader G., Molina Bonilla P., Naughton K., Maher L., Casburn M., Arndt S., Mannion A. (2021). Complex Comorbid Presentations Are Associated with Harmful Behavior Problems among Children and Adolescents with Cerebral Palsy. Dev. Neurorehabil..

[21] Leader G., Mooney A., Chen J.L., Whelan S., Naughton K., Maher L., Mannion A. (2022). The Co-Occurrence of Autism Spectrum Disorder and Cerebral Palsy and Associated Comorbid Conditions in Children and Adolescents. Dev. Neurorehabil..

[22] Casseus M., Cheng J. (2021). Children with Cerebral Palsy and Unmet Need for Care Coordination. J. Dev. Behav. Pediatr..

[23] De Clercq L.E., Soenens B., Dieleman L.M., Prinzie P., Van der Kaap-Deeder J., Beyers W., De Pauw S.S.W. (2022). Parenting and Child Personality as Modifiers of the Psychosocial Development of Youth with Cerebral Palsy. Child Psychiatry Hum. Dev..

[24] Chen Q., Chen M., Bao W., Strathearn L., Zang X., Meng L., Xu G. (2024). Association of Cerebral Palsy with Autism Spectrum Disorder and Attention-Deficit/Hyperactivity Disorder in Children: A Large-Scale Nationwide Population-Based Study. BMJ Paediatr. Open.

[25] Whitney D.G., Peterson M.D., Warschausky S.A. (2019). Mental Health Disorders, Participation, and Bullying in Children with Cerebral Palsy. Dev. Med. Child Neurol..

[26] Casseus M., Cheng J., Reichman N.E. (2024). Clinical and Functional Characteristics of Children and Young Adults with Cerebral Palsy and Co-Occurring Attention-Deficit/Hyperactivity Disorder. Res. Dev. Disabil..

[27] Cummins D., Kerr C., McConnell K., Perra O. (2021). Risk Factors for Intellectual Disability in Children with Spastic Cerebral Palsy. Arch. Dis. Child.

[28] Olusanya B.O., Gladstone M., Wright S.M., Hadders-Algra M., Boo N.-Y., Nair M.K.C., Almasri N., Kancherla V., Samms-Vaughan M.E., Kakooza-Mwesige A. (2022). Cerebral Palsy and Developmental Intellectual Disability in Children Younger than 5 Years: Findings from the GBD-WHO Rehabilitation Database 2019. Front. Public Health.

[29] Fitneva S.A., Corbett B.A., Prasad A.N. (2023). Psychosocial Correlates of Neurodevelopmental Disabilities in 2- to 3-Year-Olds. Epilepsy Behav..

[30] McMahon J., Harvey A., Reid S.M., May T., Antolovich G. (2020). Anxiety in Children and Adolescents with Cerebral Palsy. J. Paediatr. Child Health.

[31] Salie R., Eken M.M., Donald K.A., Fieggen A.G., Langerak N.G. (2022). Pain, Health-Related Quality of Life, and Mental Health of Adolescents and Adults with Cerebral Palsy in Urban South Africa. Disabil. Rehabil..

[32] Testani D., McMorris C.A., Clark C.A., Sanguino H., Condliffe E.G., Noel M.E., Kopala Sibley D.C., Brunton L.K. (2024). Investigating Physiological Symptoms Associated with Mental Health Symptoms in Youth with Cerebral Palsy: An Observational Study. Res. Dev. Disabil..

[33] Gardiner E., Miller A.R., Lach L.M. (2020). Topography of Behavior Problems among Children with Neurodevelopmental Conditions: Profile Differences and Overlaps. Child Care Health Dev..

[34] Horwood L., Li P., Mok E., Oskoui M., Shevell M., Constantin E. (2019). Behavioral Difficulties, Sleep Problems, and Nighttime Pain in Children with Cerebral Palsy. Res. Dev. Disabil..

[35] Laporta-Hoyos O., Pannek K., Pagnozzi A.M., Whittingham K., Wotherspoon J., Benfer K., Fiori S., Ware R.S., Boyd R.N. (2022). Cognitive, Academic, Executive and Psychological Functioning in Children with Spastic Motor Type Cerebral Palsy: Influence of Extent, Location, and Laterality of Brain Lesions. Eur. J. Paediatr. Neurol..

[36] de Freitas Feldberg S.C., da Silva Gusmão Cardoso T., Santos F.H., Muszkat M., Bueno O.F.A., Berlim de Mello C. (2021). Numerical Cognition in Children with Cerebral Palsy. Res. Dev. Disabil..

[37] Jarl J., Alriksson-Schmidt A. (2021). School Outcomes of Adolescents with Cerebral Palsy in Sweden. Dev. Med. Child Neurol..

[38] Micheletti S., Galli J., Vezzoli M., Scaglioni V., Agostini S., Calza S., Merabet L.B., Fazzi E. (2024). Academic Skills in Children with Cerebral Palsy and Specific Learning Disorders. Dev. Med. Child Neurol..

[39] Pereira A., Rosário P., Lopes S., Moreira T., Magalhães P., Núñez J.C., Vallejo G., Sampaio A. (2019). Promoting School Engagement in Children with Cerebral Palsy: A Narrative Based Program. Int. J. Environ. Res. Public Health.

[40] Wotherspoon J., Whittingham K., Sheffield J., Boyd R.N. (2024). Randomised Controlled Trial of an Online Cognitive Training Program in School-Aged Children with Cerebral Palsy. Res. Dev. Disabil..

[41] Ahn B., Joung Y.-S., Kwon J.-Y., Lee D.I., Oh S., Kim B.-U., Cha J.Y., Kim J.-H., Lee J.Y., Shin H.Y. (2021). Effects of Equine-Assisted Activities on Attention and Quality of Life in Children with Cerebral Palsy in a Randomized Trial: Examining the Comorbidity with Attention-Deficit/Hyperactivity Disorder. BMC Pediatr..

[42] Alibakhshi H., Siminghalam M., Avaz K.A., Salmani M., Pahlevanian A., Motaharinezhad F., Kanani Z. (2023). The Impact of Teaching Communication Skills to Mothers on Reducing Behavioral Problems in Children with Cerebral Palsy: A Quasi-Experimental Study. J. Rehabil. Sci. Res..

[43] Chen Y.-C., Chang W.-P., Liang K.-J., Chen C.-L., Chen H.-Y., Chen S.-P., Chan P.-Y.S. (2024). The Effects of Neurofeedback Training for Children with Cerebral Palsy and Co-Occurring Attention Deficits: A Pilot Study. Child Care Health Dev..

[44] Mak C., Whittingham K., Cunnington R., Chatfield M., Boyd R.N. (2022). Six-Month Follow-up of a Mindfulness Yoga Program, MiYoga, on Attention, Executive Function, Behaviour and Physical Outcomes in Cerebral Palsy. Disabil. Rehabil..

[45] Ödek U., Özcan K., Özyurt G., Akpinar S. (2022). Psychological Benefits of Equine-Facilitated Activities for Children Diagnosed with Cerebral Palsy. Sport Mont..

[46] Samijn B., Van den Broeck C., Plasschaert F., Pascal A., Deschepper E., Hoebeke P., Van Laecke E. (2022). Incontinence Training in Children with Cerebral Palsy: A Prospective Controlled Trial. J. Pediatr. Urol..

[47] Shahriari Y., Ghasemzadeh S., Vakili S. (2019). The Effectiveness of Child-Centred Play Therapy on Internalization and Extrapolation Behavioral Problems in Children with Cerebral Palsy. Iran. J. Psychiatry Clin. Psychol..

[48] Whittingham K., Sheffield J., Mak C., Wright A., Boyd R.N. (2022). Parenting Acceptance and Commitment Therapy: An RCT of an Online Course with Families of Children with CP. Behav. Res. Ther..

[49] Levy-Zaks A., Pollak Y., Ben-Pazi H. (2014). Cerebral Palsy Risk Factors and Their Impact on Psychopathology. Neurol. Res..

[50] McMorris C.A., Lake J., Dobranowski K., McGarry C., Lin E., Wilton D., Lunsky Y., Balogh R. (2021). Psychiatric Disorders in Adults with Cerebral Palsy. Res. Dev. Disabil..

[51] Whitney D.G., Warschausky S.A., Ng S., Hurvitz E.A., Kamdar N.S., Peterson M.D. (2019). Prevalence of Mental Health Disorders among Adults with Cerebral Palsy. Ann. Intern. Med..

[52] Weber P., Bolli P., Heimgartner N., Merlo P., Zehnder T., Kätterer C. (2016). Behavioral and Emotional Problems in Children and Adults with Cerebral Palsy. Eur. J. Paediatr. Neurol..

[53] Salari N., Ghasemi H., Abdoli N., Rahmani A., Shiri M.H., Hashemian A.H., Akbari H., Mohammadi M. (2023). The Global Prevalence of ADHD in Children and Adolescents: A Systematic Review and Meta-Analysis. Ital. J. Pediatr..

[54] Salari N., Rasoulpoor S., Rasoulpoor S., Shohaimi S., Jafarpour S., Abdoli N., Khaledi-Paveh B., Mohammadi M. (2022). The Global Prevalence of Autism Spectrum Disorder: A Comprehensive Systematic Review and Meta-Analysis. Ital. J. Pediatr..

[55] Reid S.M., Meehan E.M., Arnup S.J., Reddihough D.S. (2018). Intellectual Disability in Cerebral Palsy: A Population-Based Retrospective Study. Dev. Med. Child Neurol..

[56] Türkoğlu G., Türkoğlu S., Çelık C., Uçan H. (2017). Intelligence, Functioning, and Related Factors in Children with Cerebral Palsy. Arch. Neuropsychiatry.

[57] Stadskleiv K. (2020). Cognitive Functioning in Children with Cerebral Palsy. Dev. Med. Child Neurol..

[58] Brossard-Racine M., Hall N., Majnemer A., Shevell M.I., Law M., Poulin C., Rosenbaum P. (2012). Behavioural Problems in School Age Children with Cerebral Palsy. Eur. J. Paediatr. Neurol..

[59] Cobham V.E., Hickling A., Kimball H., Thomas H.J., Scott J.G., Middeldorp C.M. (2020). Systematic Review: Anxiety in Children and Adolescents with Chronic Medical Conditions. J. Am. Acad. Child Adolesc. Psychiatry.

[60] Frampton I., Yude C., Goodman R. (1998). The Prevalence and Correlates of Specific Learning Difficulties in a Representative Sample of Children with Hemiplegia. Br. J. Educ. Psychol..

[61] Silberg T., Kapil N., Caven I., Levac D., Fehlings D. (2023). Cognitive Behavioral Therapies for Individuals with Cerebral Palsy: A Scoping Review. Dev. Med. Child Neurol..

